# Cytokine-Mediated Crosstalk between Immune Cells and Epithelial Cells in the Gut

**DOI:** 10.3390/cells10010111

**Published:** 2021-01-09

**Authors:** Mousumi Mahapatro, Lena Erkert, Christoph Becker

**Affiliations:** Department of Medicine 1, Friedrich-Alexander-University Erlangen-Nuremberg, 91052 Erlangen, Germany; mousumi.mahapatro@uk-erlangen.de (M.M.); lena.erkert@uk-erlangen.de (L.E.)

**Keywords:** cytokines, signaling, inflammation, immunity, gut homeostasis, IBD

## Abstract

Cytokines are small proteins that are secreted by a vast majority of cell types in the gut. They not only establish cell-to-cell interactions and facilitate cellular signaling, but also regulate both innate and adaptive immune responses, thereby playing a central role in genetic, inflammatory, and infectious diseases of the gut. Both, immune cells and gut epithelial cells, play important roles in intestinal disease development. The epithelium is located in between the mucosal immune system and the gut microbiome. It not only establishes an efficient barrier against gut microbes, but it also signals information from the gut lumen and its composition to the immune cell compartment. Communication across the epithelial cell layer also occurs in the other direction. Intestinal epithelial cells respond to immune cell cytokines and their response influences and shapes the microbial community within the gut lumen. Thus, the epithelium should be seen as a translator or a moderator between the microbiota and the mucosal immune system. Proper communication across the epithelium seems to be a key to gut homeostasis. Indeed, current genome-wide association studies for intestinal disorders have identified several disease susceptibility loci, which map cytokine signatures and their related signaling genes. A thorough understanding of this tightly regulated cytokine signaling network is crucial. The main objective of this review was to shed light on how cytokines can orchestrate epithelial functions such as proliferation, cell death, permeability, microbe interaction, and barrier maintenance, thereby safeguarding host health. In addition, cytokine-mediated therapy for inflammation and cancer are discussed.

## 1. Introduction

The intestinal tract is a complex organ of the human body and multitasks a variety of functions, which include nutrient absorption, digestion, waste excretion, barrier regulation, and host–microbiome interactions. The majority of the gut tissue is comprised of epithelial cells and immune cells, making it the largest immune organ of the human body. Communication between these two cell types is crucial for maintaining homeostasis and responding to external threats that can lead to disease [1].

Intestinal epithelial cells (IECs) form a single strong monolayer that barricades the luminal and microbial contents from the resident immune cells. IECs have a very short life-span of about 4–5 days, after which they are shed at the villus tip and are renewed from the crypt bottom in a tightly controlled manner. However, this single epithelial cell layer is more complex in its composition and dynamic in its functions, a property that is essential for regulating physiology and pathophysiology of the gut. The majority of IECs are absorptive enterocytes followed by specialized cell types like antimicrobial peptide-producing Paneth cells (restricted to the small intestine only), mucus-producing goblet cells, hormone-secreting enteroendocrine cells, M (microfold)-cells, and tuft cells. All IEC cell types originate from *Lgr5+* stem cells that reside deep in the crypts of the large and small intestine. IECs regularly interact with neighboring immune cells and are, similar to immune cells, able to secrete molecules like cytokines, chemokines, and growth factors [1].

Intestinal immune cells are mostly reserved in the gut-associated lymphoid tissues like Peyer’s patches, lymphoid follicles, and cryptopatches. However, a diverse array of immune cells are also present in the lamina propria (LP) or intermingled in the monolayer of epithelial cells. The LP contains cells of both, the innate and the adaptive immune system, like macrophages, dendritic cells, mast cells, T-cells, B-cells, innate lymphoid cells (ILC), etc., whereas the epithelium, in contrast, only predominantly harbors T-cells (intraepithelial lymphocytes (IELs)). The spatial orientation of epithelial cells allows antigen recognition on its apical side to sense danger signals and transmit this information to the underlying LP immune cells that respond by secreting cytokines in order to restore the balance in the intestine [2]. Nonetheless, these cytokines can act positively or negatively, e.g., macrophages can signal IL-10 during colonic damage that helps in restoration of homeostasis, but also IL-1β or TNF-α, which can contribute to an enhancement of intestinal inflammation. Additionally, there are many conflicting data in the literature that show specific cytokines like IL-33 have both pro- and anti-inflammatory properties. Besides their role in inflammation, gut cytokines also participate in cell proliferation, cell death, safe-guarding tight junctions, and recruitment of immune cells to the site of infection [3]. Moreover, recent in vitro advancements in organoid culture have made it feasible to study the precise interaction of specific cytokines with epithelial cells as opposed to the old-fashioned method of using 2D cell lines. The aim of this review was to discuss recent advances in intestinal immune–epithelial crosstalk and to shed light on the complex interplay of cytokines and how they respond to threats during health and disease, with a prospect of targeting cytokines as novel treatment.

## 2. Tranquility in the Gut: Cytokines Maintaining Homeostasis

Intestinal homeostasis is maintained by a complex interplay among the epithelium, immune factors and the microbial flora. Cytokines support intestinal homeostasis by governing key cellular processes like cell death, proliferation, molecular transport, and inflammatory responses against pathogens [4]. Constitutively active cytokines that regularly shuttle among different compartments of the intestinal mucosa can modulate the division of epithelial cells and assign appropriate immune cells to establish feedback loops.

**Figure 1:**
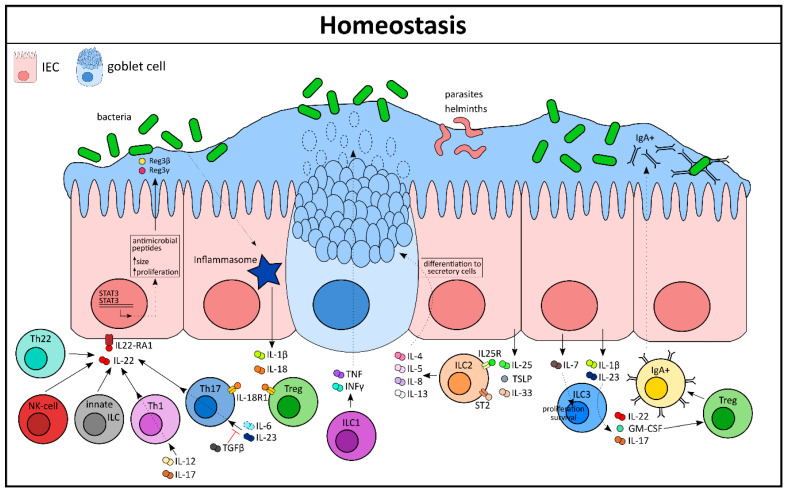
Immune–epithelial crosstalk as a guardian of intestinal homeostasis. GM-CSF, granulocyte-macrophage colony-stimulating factor; IEC, intestinal epithelial cell; ILC, innate lymphoid cell; NK-cell, natural killer cell; STAT, signal transducers and activators of transcription; Th, T helper cell; Treg, regulatory T-cell; TSLP, thymic stromal lymphopoietin.

Cytokines from the IL-1 family, like IL-1β and IL-18, play a crucial role in maintaining homeostatic conditions in the intestine (Figure 1) [5]. The release of these two cytokines is dependent on the activation of the inflammasome complex, whereas IL-1β secretion is stimulus-driven and IL-18 is constitutively expressed by the intestinal epithelium [6]. Nonetheless, a strict equilibrium of the IL-18 level is important for epithelial integrity as overexpression of IL-18 in this compartment leads to a loss of matured goblet cells and increased susceptibility to experimental colitis. On the other hand, deletion of IL-18 in IECs confers protection against DSS-induced colonic inflammation [6]. Interestingly, epithelial-derived IL-18 can directly act on IL-18R1-expressing CD4+ Th17 cells as well as Foxp3+ regulatory T-cells (Tregs) and can control their differentiation during both homeostasis and inflammation [7]. In this case, IL-18 portrays a perfect example of cytokine-mediated crosstalk, where the intestinal epithelium educates immune cells for the necessary action. Vice versa, IL-22, a member of the IL-10 family, has been shown to be primarily secreted from immune cells and can directly affect the intestinal epithelium via its receptors [8]. IL-22 is secreted from T-cells and innate lymphoid cells (ILCs). Tissue resident Th1 and Th17 cells can secrete IL-22 under the influence of IL-6 and IL-23, which on the other hand can be inhibited by TGF-β [9,10,11]. Additionally, IL-12 and IL-17 can also induce Th1 cells to secrete IL-22. Other sources of IL-22 include Th22 cells, CD8+ T-cells, γδ T-cells, and NKT cells under homeostatic and inflammatory conditions, e.g., during *Citrobacter rodentium* infection [12]. These cell populations are in close contact with the IECs that abundantly express the receptor for IL-22 (IL-22RA1) [13]. Indeed, stimulation of IL-22 on intestinal epithelial derived organoids leads to an increase in their size and proliferation rate [13]. This was supported in a study by *Zwarycz* et al. using IL-22 transgenic mice, which also showed an increased number of Ki67+ IECs and increased crypt length [14]. Mechanistically, IL-22 activates STAT3 signaling that promotes epithelial cell survival during bacterial infection by secretion of antimicrobial peptides like Reg3β and Reg3γ [15]. Thus, IL-22 plays a key role in the regulation of mucosal homeostasis.

Among the innate cells of the LP, ILCs act as first responders during tissue injury. The ILCs exhibit a similar phenotype and function as T-lymphocytes, but lack the lineage markers expressed by T-cells. They have been shown to play a crucial role in the maintenance of tissue homeostasis by several independent research groups (reviewed extensively in [16]). ILCs mediate their actions primarily by fine tuning cytokine networks between the innate and adaptive immune system. ILCs arise from the common lymphoid progenitor and categorize into three major subsets: ILC1, ILC2, and ILC3, which mirror phenotypically to Th1, Th2, and Th17 lymphocytes, respectively [17]. In humans, ILCs are distributed heterogeneously across the gastrointestinal tract with a gradual increase in their frequency from the proximal to the distal intestine, indicating their functional properties as tissue specific [18]. The T-bet expressing ILC1 population has been shown to be the main producer of TNF and IFN-γ in an immune defense against *Salmonella enterica* or *Toxoplasma gondii* infection (Figure 1). Moreover, ILC1s drive secretion of mucus from goblet cells, thereby restoring the epithelial barrier [19,20].

ILC2s are primarily involved in controlling parasitic infections in the gut and serve as an excellent example of crosstalk between epithelial and innate immune cells. During infection, parasites and helminths are in close contact with epithelial cells and elicit the secretion of a copious amount of certain cytokines like IL-25, IL-33, and TSLP (Figure 1). These epithelial-derived cytokines can directly signal to the ILC2 population that bears its receptors (IL-25R and ST2) to secrete type 2 family cytokines like IL-5, IL-13, IL-4, and IL-9. In turn, these cytokines have the ability to reorganize the epithelial cell compartment by inducing differentiation of progenitor cells to cells of the secretory lineage like the goblet cells [16]. A high number of mucus-producing goblet cells helps in the eradication of the parasite from its host. This positive feedback loop amplifies the type 2 immune response to establish homeostasis, which is mediated exclusively by cytokines. ILC2s also respond to eicosanoids, such as prostaglandin D2 (PGD2) and leukotriene D4, to secrete type 2 cytokines. However, this finding has been mostly studied in inflamed lung tissue. A similar working mechanism has yet to be elucidated in the gut [21]. Although much less is known about human ILC2s, they have been found in many different places such as in the peripheral blood, fetal tissues, and the adult gut [22].

ILC3s, together with lymphoid tissue inducer (Lti) cells, are vital for the organogenesis of secondary lymphoid organs during fetal development and are identified by the expression of the surface receptor RORγt. They are abundantly present in the gastrointestinal tract and are, notably, the most characterized ILC population to take part in intestinal homeostasis. It has been implicated that IL-7 can modulate the survival and proliferation of ILC3 by increasing the expression of RORγt [23,24]. In fact, studies with IL-7 reporter mice have confirmed that it can be secreted by EpCAM+ IECs and this expression is controlled by the commensal microbiota of the healthy gut [25]. Other than that, IL-1β and IL-23 have been shown to be robust stimulators for ILC3, compelling the secretion of effector cytokines like IL-22, GM-CSF, and IL-17 [26]. ILC3-derived IL-22 plays a major role in mounting an innate response and reshaping the microbiota after a pathogenic invasion of bacteria like *Citrobacter rodentium* and *Clostridium difficile* [27,28]. Similarly, ILC3-derived IL-17 has been shown to be crucial against opportunistic pathogens like *Candida albicans* in the oral mucosa. Indeed, mice lacking the ILC3 population are more susceptible to bacterial infections and are often rendered incapable of controlling a pathogen invasion, possibly due to the fact that in these mice the IECs have an impaired antimicrobial peptide production [16]. GM-CSF, another important regulator secreted by ILC3, acts like a cytokine and controls intestinal Tregs. Intestinal Tregs are crucial for homeostasis as they control effector cell responses in the gut as well as the selection of immunoglobulin A (IgA) plasma cells, thereby supporting tissue repair [29].

Additionally, ILC3 can directly mediate fucosylation of IECs by inducing expression of intestinal epithelial enzymes like Fut1 and Fut2 (fucosyltransferase enzymes). This is an important mechanism that enables IECs to survive harmoniously within the commensals [30]. Collectively, it is clear that even under homeostatic conditions there is constant crosstalk between the immune and the epithelial cells mediated by a composed cytokine network (Figure 1).

## 3. Intestinal Microbiota Influences Cytokine Production

**Figure 2:**
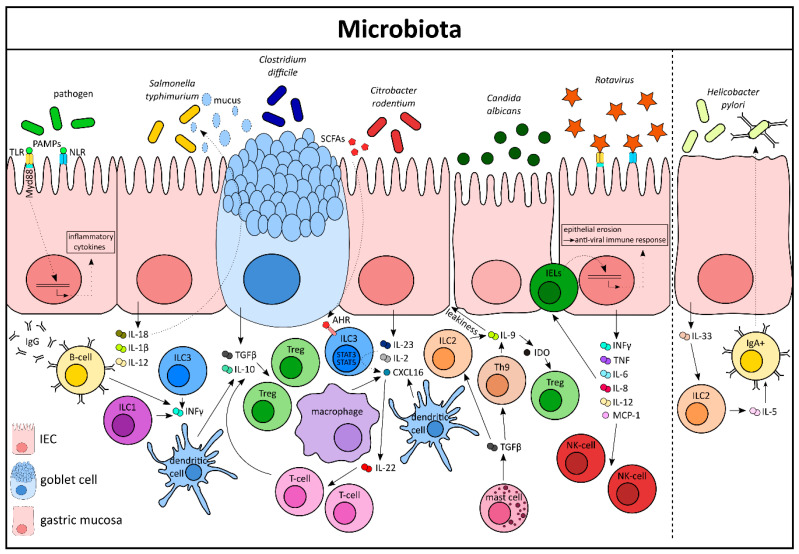
Cytokine signaling between intestinal epithelial cells and immune cells regulate the host response to infections. AHR, Aryl hydrocarbon receptor; IDO, indolamin-2,3-dioxygenase; IELs, intraepithelial lymphocytes; MCP-1, monocyte chemoattractant protein-1; NLR, NOD-like receptor; PAMPs, pathogen-associated molecular patterns; SCFAs, short-chain fatty acids; TLR, Toll-like receptor.

A diverse microbiome in the intestine is essential for maintaining a robust immune system and homeostasis at the epithelial barrier. The host and the microbial commensals have a symbiotic relationship, where the host provides the required nutrients to facilitate microbial survival and the microbes secrete components that not only prevent colonization of extrinsic pathogens but also support intestinal repair through the promotion of cellular proliferation and differentiation [31]. Changes in the microbial composition induced by diet, alcohol, antibiotics intake, or pre-existing genetic aberrations can lead to dysbiosis in the gut and deregulation of cytokine signatures. A functional consequence of dysbiosis is the initiation of immune-related diseases, including inflammatory bowel diseases (IBD) [32]. Microbes interact with IECs via pattern recognition receptors (PRRs), like Toll-like receptors (TLRs) [33], and its adaptor myeloid differentiation primary response protein 88 (MYD88) [34] and NOD-like receptors (NLRs) [35,36], NOD-, LRR-, and pyrin domain-containing 6 (NLRP6) (Figure 2) [36]. Of note, NLRP6 deficiency has been shown to induce an altered microbiota, reduction in levels of IL-18, intestinal hyperplasia, and colonic enteritism underlining the crucial relationship between the microbiota and homeostasis [37,38].

The complex microbiota is an amalgamation of a multispecies community comprising of bacteria, fungi, viruses, archaea, and protozoans. After a bacterial invasion, a complex cascade of signals initiate the release of cytokines, chemokines, acute phase proteins and other effectors of the humoral immunity [39]. For example, IL-18 functions as a pleiotropic cytokine that can induce enterocytes to produce more AMPs and goblet cells to produce more mucus in order to eradicate pathogens like *Salmonella Typhimurium* (Figure 2) [40]. A recent interesting study linked IL-18 to the microbiota and the central nervous system by showing enteric neurons to be the major producers of this cytokine, other than IECs and immune cells [41]. *Salmonella* gastroenteritis infection results in the release of IL-1β and IL-12 that can induce ILC1s and B-cells to secrete high levels of IFN-γ and IgG antibodies, respectively [19,42]. Additionally, NCR3+ (natural cytotoxicity receptor) ILC3s have been shown to play a crucial role in both *Salmonella* and *Listeria monocytogenes* infections, in a Runt-related transcription factor 3 (Runx3)-dependent manner, modulating IFN-γ secretion [43]. Certain bacterial species from the *Clostridiales* family, like *Clostridium difficile*, can educate goblet cells and CD103+ dendritic cells (DCs) to secrete TGF-β and IL-10, thereby generating ample signals to increase the Treg population. DCs and T-cells can also sense *Clostridium*-derived metabolites, like short chain fatty acids (SCFA) and tryptophan, to induce proliferation of Tregs [44]. However in Rag1^−/−^ mice that lack T- and B lymphocytes, the ILC3s are the major responders during *Clostridium* infection, producing IL-22, IL-33, TNF, Nos2, IL-17a, and Reg3γ [42]. Indeed, ILCs and natural killer (NK) cells show high levels of plasticity when it comes to tackling certain bacterial infections like *Citrobacter rodentium,* a mouse-specific pathogen that mimics the disease symptoms caused by the human pathogens enteropathogenic *Escherichia coli* (EPEC) and enterohaemorrhagic *Escherichia coli* (EHEC). Both ILC1s and NK cells promote bacterial clearance by producing IFN-γ, TNF, and IgG in the colon, which contribute to homing of CD4+ T-cells to the intestine [45]. However, in the initial phase of a *Citrobacter* infection, IL-22 producing ILC3s are activated via their surface receptors (aryl hydrocarbon receptor (AHR), RORγt, Vitamin A and D receptors) or bacteria derived metabolites (SCFAs, Butyrate), which eventually leads to recruitment of T-cells and subsequent bacterial clearance [46,47,48,49]. Additionally, crosstalk among myeloid cells like CX3CR1+ DCs, TL1A+ macrophages, and ILC3s mediates the release of the chemokine CXCL16 and controls the expression of IL-22 and antimicrobial peptides (AMPs) [50,51,52]. Mechanistically, STAT5a, STAT5b, and STAT3 signaling, initiated by IL-2 or IL-23, respectively, in ILC3 has been shown to play a major role in *Citrobacter*-mediated colitis [53]. In contrast, infection with the stomach residing pathogen *Helicobacter pylori* can induce Tregs and ILC2s to elicit a specific TCR repertoire that drives T-cell polarization [44]. Indeed, infection with *Helicobacter* leads to the release of IL-33 from gastric mucosa and subsequent activation of ILC2s, which can secrete IL-5 and enhance the IgA release by B-cells [54,55,56]. This mechanism partially contributes to protection against the infection [57].

Fungi, like *Candida albicans*, are part of the human gastrointestinal tract microbiota. However, excessive colonization of *Candida* in the gut can lead to unspecific chronic disorders like fatigue, diarrhea, allergies, and skin irritation [58]. A recent study showed IL-9 and mast cells to be key players in *Candida* pathogenesis (Figure 2). TGF-β released from mast cells activates Th9 cells and ILC2s to secrete IL-9, which pivotally contributes to immune tolerance via the indoleamine 2, 3-dioxygenase enzyme (IDO). However, *Candida*-driven IL-9 also leads to a disruption of the epithelial barrier and increases leakiness in the gut, leading to inflammation [59]. Of note, differences in the gut fungal microbiota composition are associated with mucosal inflammation and disease activity in IBD patients [60].

Interferons (IFN) are critical mediators for antimicrobial host responses. Type II IFNs (IFN-γ) are mostly associated with providing protection against a broad range of intracellular microorganisms, whereas Type I (IFN-α, IFN-β) and type III (IFN-λ1, -λ2, and -λ3) IFNs primarily mediate antiviral responses. IFN receptors (IFNR) are highly expressed by IECs and receptor–ligand interaction leads to activation of the JAK/STAT pathway and expression of IFN-stimulated genes [61]. Viruses, like Rotavirus, are a common cause for gastroenteritis and exhibit strong epithelial aberrations in the small intestine of infected patients [62]. In fact, mice deficient for IFNR in the IEC compartment (*Ifnlr1^-/-^* mice) are more susceptible to viral infections [63,64]. On a mechanistic level, Rotavirus activates PRRs expressed by both IECs and DCs, like Toll-like receptor 3 (TLR3), Retinoic acid-inducible gene-I (RIG-I), and melanoma differentiation-associated gene-5 (MDA-5), which leads to the secretion of proinflammatory cytokines like IFN, TNF, IL-6, IL-8, IL-12, and MCP-1. These aforementioned cytokines lead to collateral damage by recruiting cells of both innate and adaptive immunity, most notably CD3+NK1.1+CD8αα+ IELs, which, in turn, results in epithelial damage and mucosal erosion followed by an antiviral state in IECs [65] (Figure 2).

Hence, a comprehensive study of the microbial composition, microbe-derived metabolites, host factors interacting with the microbiota, and the underlying complex interactions with immune and epithelial cells is vital for analyzing multiple aspects of intestinal diseases.

## 4. Cytokine Regulation in Intestinal Pathology

Intestinal diseases can affect any part of the gut, starting from the proximal duodenum through to the rectum. It is associated with many complications, like constipation, irregular bowel movement, or abdominal pain, that compromise the overall health and lifestyle of an individual. Etiological studies have identified common factors like increased epithelial permeability, dysbiosis, inflammation, visceral hypersensitivity, immune cell infiltration, and altered brain–gut interactions. Prevalent gastrointestinal diseases are IBS (irritable bowel syndrome), Celiac disease, and IBD (inflammatory bowel diseases) [66].

### 4.1. Irritable Bowel Syndrome and Celiac Disease

IBS is a gastrointestinal disorder with a global prevalence of 11.2% [67]. Clinical symptoms of IBS mimic other bowel diseases and are associated with abdominal pain or discomfort, stool irregularities, and bloating [68]. The systematic circulating cytokine profile of patients suffering from IBS has been studied, but the implications of these findings on mucosal immunity remain inconclusive. For example, in vitro experiments using peripheral blood mononuclear cell (PBMC) cultures from IBS patients showed a reduced secretion of IL-10 [69,70], an anti-inflammatory cytokine, and an elevated level of proinflammatory cytokines like IL-1β, IL-6, IL-8, IL-12, and TNF at steady state conditions [69,70,71]. Additionally, in some studies, cytokines such as chemokine (C-C motif) ligand-16 (CCL-16) [72], macrophage migration inhibitory factor (MIF), and monocyte chemotactic protein-1 (MCP1 or CCL2) [73] were found to be elevated in the serum of IBS patients compared with controls. In fact, polymorphisms in genes encoding inflammatory cytokines have been reported in IBS patients [74], including SNPs in the IL-6 region, which occur during the progression of the disease, and indicate an increased risk with cytokine dysfunction [75]. Unfortunately, at present, little is known about how these findings translate to immune–epithelial communication in the gut, and further mechanistic studies would be required to better understand the pathophysiology of IBS.

Celiac disease is an autoimmune disorder of the gut that primarily affects the small intestine and is induced by the ingestion of gluten in the diet [76]. The genetic predisposition to this protein that can elicit an intestinal enteropathy is still unknown. IECs are the first cells to be in contact with the gluten or gluten-derived components like gliadin and are responsible for mounting an adaptive immune reaction at the effector site (e.g., the lamina propria) by secreting IL-15 [77]. IL-15 alters epithelial barrier functions by disrupting the tight junction protein Zonulin [78], thereby increasing permeability [79,80]. Subsequently, IL-15 can also induce IELs to secrete IFN-γ, which can activate T-cells like CD4+ α/β and CD8+ α/β cells to acquire an aberrant NK-like phenotype that can kill enterocytes in a T-cell receptor (TCR)-independent manner. This unique pathway facilitates an increased gluten/gliadin uptake that ultimately results in villous atrophy in the mucosa of celiac disease patients [81,82]. IECs also secrete transglutaminase 2 (TG2) and autoantibodies generated against this enzyme can be detected in celiac patients. Autoantibody levels are positively correlated with the disease severity [83]. In summary, the cytokine profile in this disease is predominantly proinflammatory and a failure to control the inflammatory response may be one of the factors underlying gluten intolerance in genetically predisposed individuals [84,85].

### 4.2. Inflammatory Bowel Diseases

The etiology of inflammatory bowel diseases (IBDs) still remains unknown, but there has been mounting evidence that it is associated with an abnormal microflora, unregulated immune responses, genetic susceptibility, and lifestyle choices. IBD is broadly categorized into Crohn’s disease (CD) and ulcerative colitis (UC). In UC, inflammation is mostly confined to the colon, whereas in CD it can affect many parts of the gastrointestinal tract, often leading to strictures and fistulas. Extraintestinal manifestations, like joint pain, psoriasis, ankylosing spondylitis, or primary sclerosing cholangitis (PSC), are frequently diagnosed in both forms of IBD [16,86]. Genome-wide association studies (GWAS), mouse models of colitis, and in vitro organoid experiments from murine and human gut tissue have greatly advanced the understanding of the molecular mechanisms involved in cytokine signaling and immune–epithelial interactions and their impact on mucosal inflammation [87]. In the present scenario, it is clear that a perturbed cytokine cascade amplifies inflammation.

**Figure 3:**
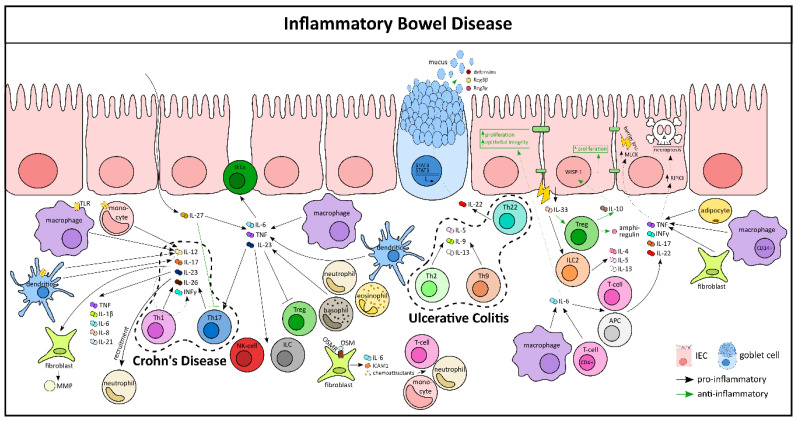
Cytokine-mediated immune–epithelial crosstalk in inflammatory bowel diseases. APC, antigen-presenting cell; ICAM, intercellular adhesion molecule-1; MLCK, myosin light chain kinase; MMP, matrix metalloproteinase; OSM, Oncostatin M; OSMR, Oncostatin M receptor; RIPK3, receptor interacting serine/threonine kinase 3; WISP-1, WNT 1-inducible signaling pathway protein 1.

On a cellular level, activated lamina propria cells secrete a plethora of cytokines at the local site of inflammation, including proinflammatory cytokines (TNF, IFN-γ, IL-6, IL-12, IL-21, IL-23, IL-17, integrins, etc.), as well as anti-inflammatory cytokines (IL-10, TGFβ, IL-35, etc.) [88]. CD is usually designated as a type 1 driven disease with an elevated production and activation of Th1 and Th17 cells and their cytokines like IL-12, IL-23, IFN-γ, and IL-17. In contrast, UC has been associated with a type 2-like inflammation, as indicated by an increased production of Th2 and Th9 cells and cytokines like IL-13, IL-5, and IL-9 (Figure 3) [88]. Interestingly, IL-23 has been shown to be associated with both CD and UC pathology. An increased production of IL-23 by macrophages, dendritic cells, and granulocytes has been observed in various mouse models of colitis and in IBD patients. IL-23 mediates activation and cytokine production of other immune cells in colitis, such as NK cells, IELs, ILCs, and Th17, while simultaneously blocking the activation of Tregs. Genetic studies have identified single nucleotide polymorphisms in the IL-23R gene in IBD patients, suggesting that IL-23R signaling affects disease susceptibility [89]. Similar to IL-23, another cytokine from the same family, IL-12 is also implicated in IBD pathology. It is predominantly secreted by DCs, monocytes, and macrophages after pathogen recognition via Toll-like receptors. IL-12 induces the production of IFN-γ by promoting the differentiation of Th1, thereby forming a link between innate and adaptive immunity [90,91]. The role of IL-27 in IBD is more complex as it has been shown to be both pro- and anti-inflammatory in nature [92]. On one hand, exogenous administration of IL-27 via food or subcutaneous injection led to inhibition of Th17 cells and amelioration of TNBS-induced colitis, eventually decreasing the intestinal inflammation with a reduced pathologic score and inflammatory cytokines [93,94]. On the other hand, IL-27Rα^−/−^ mice developed less severe colitis after DSS treatment when compared to their wild-type controls, characterized by reduction in inflammatory cytokines (IL-6, TNF, and IFN-γ). Additionally, in another model of T-cell-mediated colitis, transfer of IL-27Rα^−/−^ T-cells resulted in diminished weight loss and reduced intestinal inflammation when compared to transferring wild-type T-cells [95].

Both IL-6 and TNF are central regulators in IBD pathogenesis. IL-6 is majorly produced by macrophages and CD4+ T-cells in the inflamed mucosa of mice and in human colons (Figure 3) [96]. IL-6 exerts its proinflammatory functions by activating multiple cells, including antigen-presenting cells (APC) and T-cells and their signature cytokines (IFN-γ, TNF, IL-17, IL-22 etc.). Besides its proinflammatory function, IL-6 has an important role in restoring homeostasis in IECs by stimulating their proliferation [96]. In fact, during an ongoing inflammation, the expansion of IECs is crucial for the intestinal barrier function as closure of the eroded area is the key to the resolution process. Hence, mucosal healing is critically dependent on cytokines released in its near vicinity, like TNF. Experimental studies have shown that membrane-bound TNF, rather than soluble TNF, has a major role in driving intestinal inflammation. TNF is mainly produced by CD14+ macrophages, adipocytes, fibroblasts, and T-cells from patients with IBD [97,98]. TNF signaling in IECs of the terminal ileum results in Paneth cell necroptosis via RIPK3 [99]. In murine models of experimental colitis, TNF signaling increased MLCK expression, resulting in tight junction dysregulation and barrier loss in the IEC compartment [100]. Of note, IL-6 and IL-23 also activate T-cells, like Th17, to produce their signature cytokines, such as IL-17A, IL-17F, and IL-26. Functionally, Th17-secreted cytokines were found to mediate proinflammatory responses, like the upregulation of TNF, IL-1β, IL-6, IL-21, and IL-8; the recruitment of neutrophils; and the secretion of matrix metalloproteinases (MMP) by intestinal fibroblasts [101,102,103]. These findings suggest that Th17-secreted cytokines contribute to tissue destruction in IBD. Interestingly, Th22 effector cells are abundant in patients with UC, but not in CD patients. These cells produce anti-inflammatory cytokines, such as IL-22, that participate in epithelial cell proliferation, wound healing, and the production of antimicrobial proteins, such as defensins and mucins, as well as REG3β and REG3γ proteins via STAT3 activation [104]. In support of a protective function of IL-22, it was demonstrated that IL-22 treatment can protect mice from T-cell-dependent colitis [105].

Another pleiotropic cytokine, IL-33, along with its receptor, ST2, has been associated with IBD risk loci genes and is shown to be upregulated in UC patients [106,107]. IL-33 is also increased in the colonic mucosa of both TNBS- and DSS-induced murine colitis. ST2^−/−^ mice exhibit reduced disease severity in colitis models and administration of exogenous IL-33 aggravates the disease. This is associated with a marked elevation of IL-4, IL-5, and IL-13; a significant reduction of IL-17 and IFN-γ; impairment of the epithelial barrier; and a delay of wound healing of the injured colonic epithelium [108,109,110,111,112] (Figure 3). In sharp contradiction, IL-33 has also been shown to protect against intestinal inflammation by activating Foxp3+ Tregs and ILC2 cells and by the production of amphiregulin [113,114]. These contrasting animal experimental results indicate the complicated role of IL-33 in IBD, and hence, require further investigation. A recent interesting study showed the cytokine oncostatin M (OSM) to be highly upregulated in inflamed mucosal tissue of IBD patients [115]. The OSM receptor (OSMR) is expressed by nonhematopoietic and nonepithelial intestinal stromal cells, which respond to OSM by producing various pro-inflammatory molecules, including IL-6, the leukocyte adhesion factor ICAM1, and chemokines that attract neutrophils, monocytes, and T-cells [116]. However, in a counter regulatory mechanism, OSM promotes epithelial repair during inflammation [117].

In recent years it has been unanimously consolidated that loss-of-function mutations in the genes encoding IL-10 and the IL-10 receptor are associated with very early onset IBD. For instance, mice deficient in the anti-inflammatory cytokines IL-2 or IL-10 develop spontaneous colitis [96]. Intestinal Tregs are the major producers of IL-10, which exerts its protective function on epithelial cells by inducing proliferation and by stimulating synthesis of Wnt1-inducible signaling protein-1 (WISP-1) (Figure 3) [118]. Interestingly, gut microbiota seemed to play a crucial role in the development of colitis in this mouse model, as germ-free IL-10^−/−^ mice did not develop colitis and the administration of antibiotics prevented colitis. Indeed, it has been narrowed down to a single species, *Helicobacter hepaticus*, which is responsible for this exacerbated disease in this model [119]. Collectively, it is now clear that cytokines have a fundamental role in IBD pathogenesis. Hence, blocking proinflammatory cytokines or using recombinant anti-inflammatory cytokines were initiated to treat patients with IBD (Figure 3).

### 4.3. Cytokine Networks in Colorectal Cancer

Colorectal cancer (CRC) is, like any other cancer, a complex disease with a high prevalence globally. Malignant epithelial cancer cells are supported by the tumor microenvironment, which includes various immune cells, cancer-associated fibroblasts, and circulating cytokines [120,121], many of which have been demonstrated to promote CRC progression [122]. Cytokines promoting tumorigenesis are IL-4, IL-6, IL-8, IL-11, IL-17A, IL-22, IL-23, IL-33, TNF-α, TGF-β, and VEGF (vascular endothelial growth factor), whereas cytokines with antitumorigenic properties are IFN-γ, IL-12, IL-15, IL-17F, and IL-18. The contribution of the cytokines IL-1, IL-9, IL-10, IL-21, and GM-CSF to CRC development and progression remains unclear [122].

**Figure 4:**
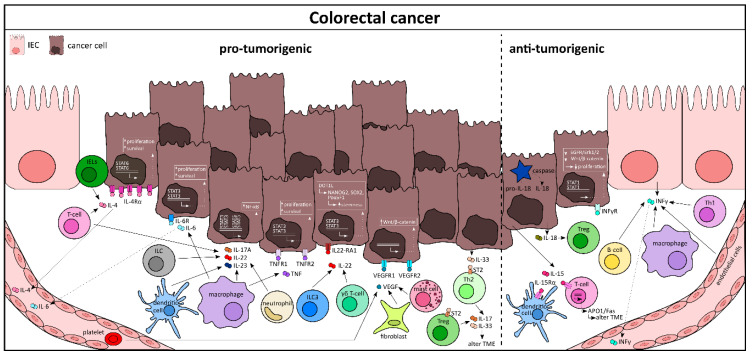
Cytokine signaling between intestinal epithelial cells and immune cells as a regulator of colorectal cancer development. TME, tumor microenvironment.

The protumorigenic role of IL-4 is evident from experimental mouse models of CRC using AOM/DSS. It has been shown that IL-4- or IL-4R-deficient mice develop far less tumors than their control counterparts [123]. However, a type 2 signature, including IL-5, IL-13, and IL-9, seems to have no prognostic advantage in CRC patients [124]. T-lymphocytes and IELs are the major source of IL-4 in cancer tissue (Figure 4) [125]. With a very high expression of IL-4Rα on malignant IECs [126], IL-4 signaling leads to phosphorylation of the signal transducer and activator of transcription 6 (STAT6) in IECs, as well as in other hematopoietic cells [127]. Of note, STAT6 phosphorylation is an essential biomarker in the prediction and prognosis of CRC patients [128]. In CRC patients with distant metastases, IL-4 serum levels were increased when compared with patients without metastases [129]. Similarly, IL-6 has also been shown to be upregulated at both serum- [129] and expression-levels in CRC tissues. In the AOM/DSS animal model, IL-6 deletion led to ameliorated tumor development. In fact, IL-6 trans-signaling is critical for IEC survival, a phenomenon that malignant cells take advantage of, thereby facilitating tumor progression [130,131]. This in turn leads to activation of STAT3, which negatively correlates with the survival of CRC patients [132]. It is noteworthy that cytokines like IL-23, VEGF, IL-17, and IL-22 work in synergy when it comes to CRC tissues [133]. IL-23 levels were enhanced in tumor tissue and IL-23a^-/-^ mice developed fewer tumors when compared to controls in an Apc^Min/+^ animal model of intestinal carcinogenesis [134]. While dendritic cells, macrophages, and neutrophils remain the main source of IL-23, once released it can exert its effect on both T-lymphocytes and ILCs to produce secondary cytokines like IL-17A and IL-22 [135,136]. These secondary cytokines, together with IL-23, can activate NF-κB, STAT3 [137], and STAT5 [138] signaling in malignant IECs. It is now well established that IL-22 supports intestinal stem cells or stemness of IECs. In a recent publication from *Gronke K* et al. it has been shown that stem cells deprived of IL-22 signaling were more likely to give rise to colon cancer when exposed to carcinogens like AOM. A possible mechanism for this observation is by escaping DDR (DNA damage response)-controlled apoptosis [139]. ILC3s and γδ T-cells are the major source of IL-22, which in turn can lead to the activation of the transcription factor STAT3 and therefore the expression of histone 3 lysine 79 (H3K79) methytransferase DOT1L. This complex induces a few stem cell genes like NANOG, SOX2, and Pou5F1, resulting in increased stemness and tumorigenic potential [140]. Likewise, in the AOM/DSS experimental model, both VEGF and its receptor VEGFR2 were strongly upregulated and treatment with anti-VEGF reduced tumor progression [141]. Similarly, inhibition of VEGFR in Apc^Min/+^ animals resulted in reduced tumor burden [142]. VEGF is known to be secreted by many cells, such as cancer-associated fibroblasts, platelets, and mast cells, and VEGFR1 signaling in IECs can activate Wnt/β-catenin signaling, an important regulator in cancer progression [143].

Colorectal cancer is sometimes the consequence of prolonged and recurrent inflammation in the gut (colitis-associated colorectal cancer or CAC), especially in UC patients [144]. The cytokine profile in these patients acts as a decisive factor in the tumor progression and metastases [145]. A few cytokines, like TNF and IL-6, have been shown to be the driving factors that bridge these two diseases (reviewed in [146]). Activated macrophages are able to produce TNF, which then binds to TNFR (both 1 and 2) expressed by IECs and other hematopoietic cells [147,148]. Activation of the TNFR leads to phosphorylation of STAT3 and/or induction of the NK-κB signaling cascade, similar to that of IL-6, which supports malignant cell survival and proliferation [149]. The deleterious effect of TNF is confirmed by using both animal models of CRC (Apc^Min/+^ and AOM/DSS), where abrogation of TNF signaling strongly diminished tumor growth [150]. Additionally, TNF has also been shown to promote tumor growth and metastasis via induction of epithelial-derived oncogene MACC1 [145]. Certain cytokines, like IL-33, a member of the IL-1 family, can regulate CRC progression by controlling the tumor microenvironment (TME). IL-33 is highly expressed by epithelial cells within adenomas and cancer cells, whereas the expression of the IL-33 receptor (ST2) can be localized predominantly on both stromal cells and epithelial cells [151]. Genetic ablation of IL-33 signaling in Apc^Min/+^ mice or inhibition by using neutralizing antibodies led to reduced proliferation, increased apoptosis and diminished angiogenesis in polyps, which overall reduced the tumor burden [152]. ST2 is also expressed by immune cells like Th2 cells [153] and Foxp3+ Tregs [154]. By releasing cytokines like IL-17 and IL-33, the recruited immune cells are able to remodel the epithelium [155] and organize the TME to promote malignancy. In addition, both IL-8 and IL-11 are also characterized as protumorigenic [122]. IL-8 mainly acts on myeloid cells and signals through its receptor CXCR1/2 to activate the Akt and MAPK pathways to promote the expression of genes responsible for cell proliferation, invasion, and angiogenesis [156]. IL-11, on the other hand, exerts its protumorigenic effects by activating TGF-β and driving a STAT3-dependent cascade to promote cancer [157,158].

One might hypothesize that the cancer progression and promotion is determined by the ratio of pro- versus antitumorigenic cytokines at a given time point. As a mechanism to counter tumor development, the host tries to eradicate the malignant cells by evoking an antitumor immune response and a wide range of cytokines with antitumor functions. For example, a higher level of IFN-γ in the serum of CRC patients is correlated with the absence of nodal metastases and thus a better prognosis (Figure 4) [159]. The protective role of IFN-γ in clinical cases is supported by results obtained from animal models of cancer, where deletion of IFN-γ (*Ifng^−/^*^−^ mice) shows a higher tumor burden when compared to controls [160]. IFN-γ is produced by many cell types in the cancer tissue, most notably the Th1 cells, B-cells, macrophages, and endothelial and epithelial cells [161]. Mechanistically, IFN-γ signaling, via its receptor (IFNγR), induces STAT1 phosphorylation and inhibits the EGFR/Erk1/2 and Wnt/β-catenin signaling pathways, thereby decreasing cell proliferation in tumor epithelial cells [161]. Similarly, IL-15 has also been implicated for its antitumorigenic role in several mouse models of cancer [162,163]. At the molecular level, IL-15 released from epithelial cells binds to IL-15Rα expressing myeloid cells (dendritic and CD8+ T-cells), which activate the APO-1/FAS- or granule-mediated cytotoxic pathway [164]. Another IL-1 superfamily cytokine, IL-18, plays a major role in gastrointestinal cancer as well. Pro-IL-18 is constitutively expressed by many cell types of the gut, including epithelial cells. Mature IL-18 is processed from its preform in a caspase-dependent manner and is secreted from the inflammasome complex [165]. IL-18 restricts Th17 differentiation and promotes the expression of effector molecules from Tregs, thereby mediating host immunity [7]. Indeed, mice deficient for IL-18 or its receptor (IL-18r1) develop more tumors upon AOM/DSS treatment, compared with wild-type controls [166]. Additionally, IL-18 and IL-12 work in synergy to activate type 1 immune responses by controlling the DNA damage protein ADP-ribosyltransferase diphteria toxin-like 1 (ARTD1) and subsequent IFN-γ production by myeloid cells [167]. Contrary to IL-17A, its family member, IL-17F, has been implicated in antitumorigenic activity. IL-17F knock out mice appear to develop more neoplastic lesions than wild-type mice upon the administration of AOM/DSS [168]. In this model IL-17F inhibited tumor angiogenesis by regulating VEGF [169]. However, a recent report highly contradicted this finding by suggesting IL-17F as an oncogene in CRC [170]. Additionally, loss of the IL-17 receptor D has been shown to promote inflammation-associated tumorigenesis [171]. At present, additional research is needed to elucidate the role of different IL-17 family cytokines in colon cancer in order to strategize therapy.

Collectively, there is no doubt that cytokines released in the TME play an essential role in CRC development and progression [172]. Identifying complex cytokine network signatures at different stages of the disease can help in designing precision medication more efficiently [173]. Combinatorial drugs that target multiple cytokines hold great promise for the future (Figure 4).

## 5. Cytokine-Mediated Therapy for Gut Diseases

As previously described, immune–epithelial cell interactions, mediated by numerous cytokines, play a crucial role in the pathogenesis of various gut diseases. Therefore, targeting of such cytokines is considered a rational approach to treating intestinal diseases.

**Figure 5:**
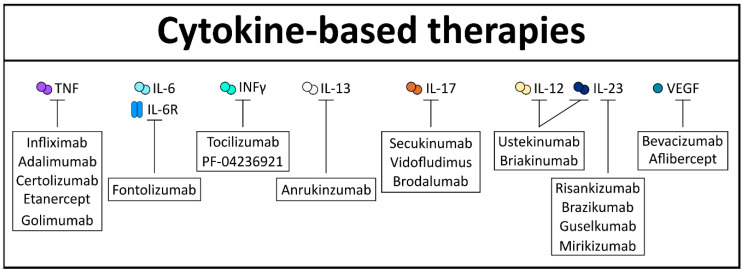
Monoclonal antibodies developed for targeting cytokines in intestinal diseases.

Existing approaches for CRC therapy are well-established adjuvants like FOLFOX4 (fluorouracil/folinic acid, oxaliplatin) and FOLFIRI (folinic acid, fluorouracil, irinotecan). Future approaches of CRC therapy might focus on the complex cytokine network in order to specifically target and inhibit cancer cells by altering the TME. One of the first FDA-approved cytokine-targeted immunotherapies in the field of CRC is bevacizumab, an anti-VEGF antibody (Figure 5). In a first study, CRC patients treated with bevacizumab showed a longer median overall survival when combined with FOLFOX4 [174] and, in a second study when it was added to chemotherapy treatment, it improved progression-free survival [175]. However, in contrast to the first study, in the second study, bevacizumab exhibited no beneficial increase of the overall survival, leading to a lot of discrepancy. Similarly, the VEGF inhibitor, aflibercept, could also show improved overall median survival in CRC patients when combined with FOLFIRI chemotherapy [176].

With regard to IBD, TNF inhibition is of especial interest, since it is considered a crucial driver of intestinal inflammation. In fact, different publications have shown the positive effect of TNF inhibition on experimental induced murine colitis [177]. One of the first-in-class TNF inhibitors approved for the treatment of IBD is infliximab, a monoclonal antibody, considered effective in both UC and CD [178,179]. Indeed, infliximab established a classical example of proof-in-concept, where blocking of a single cytokine was effective against an immune mediated disorder [180]. Later on, these beneficial effects led to the invention of various other TNF inhibitors that target the membrane-bound and/or soluble form of TNF with different affinities. Among them are adalimumab, certolizumab, etanercept, and golimumab. However, in the ensuing years, several patients failed to respond or lost the response to these inhibitors due to immunogenicity against the compound [181]. Additionally blocking of TNF signaling increases the risk for the development of infectious disease, since TNF is an important driver in pathogen clearance as well [182]. Of note, TNF blockers/ inhibitors have been shown to reduce the frequency of CAC in treated IBD patients, although bigger IBD cohorts are necessary to confirm this initial observation [183].

The limitations of TNF therapy ignited the research for finding alternative anti-cytokine therapies for intestinal diseases. In this regard, another promising target was IL-6 or its receptor IL-6R. Indeed, inhibition of this signaling cascade has been shown to effectively diminish murine induced colitis [131,184]. Clinical trials using the IL-6R inhibitor tocilizumab exhibited clinical efficacy in CD patients [185] who were unresponsive to TNF inhibition [186]. Unfortunately, IL-6 inhibition leads to severe side effects, like the development of abscesses or perforation, as demonstrated with the IL-6 inhibitor PF-04236921 [186]. Furthermore, it has been shown that IL-18 blockade, by using either neutralizing anti-murine IL-18 antiserum [187] or by the administration of an adenovirus expressing IL-18 antisense mRNA [188], diminishes inflammation in murine models of colitis. This, however, needs to be analyzed further, since no clinical trials for anti-IL-18 therapy have been initiated so far. Interestingly, IL-12 and IL-23 share the p40 subunit and targeting this subunit simultaneously has been shown to be an effective target in intestinal inflammation. Many clinical trials were performed with ustekinumab, a general p40 inhibitor, exhibiting great efficacy in CD patients, even in patients who were intolerant to TNF inhibition [189,190,191], including long-term efficacy and safety [192]. In contrast, another anti-IL12/IL-23p40 targeting antibody, called briakinumab, showed a lack of efficacy in the treatment of intestinal inflammation, probably due to increased immunogenicity when compared with ustekinumab [193]. Notably, specific neutralization of the IL-23 subunit p19 could abrogate colitis in different inflammatory animal models [194,195,196]. Recently, clinical trials supporting these observations, by using risankizumab, a selective monoclonal humanized antibody against IL-23p19, showed remission in patients with CD [197]. Furthermore, brazikumab, which also specifically inhibits IL-23p19, was tested in CD patients and could show first beneficial effects in a phase 2a clinical study [198]. Two relatively new IL-23p19-targeting biologicals are guselkumab and mirikizumab, both currently in phase 2 clinical trials for CD and UC. First results of mirikizumab in the treatment of UC are promising [199] and encourage further investigations, as cytokine-mediated therapies for UC are less common than for CD. Similarly, fontolizumab, an anti-INF-γ antibody in a phase 2 study, showed promising therapeutic effects in active CD patients by demonstrating increased rates of clinical response and remission compared with placebo [200].

However, targeting cytokines in intestinal pathogenesis has also shown some failure. Indeed, IL-13 inhibitors, like anrukinzumab, exhibited no statistically significant changes of disease severity in UC patients [201]. Comparably, anti-IL-17 therapeutics, like secukinumab or vidofludimus, have failed to reduce intestinal inflammation, and even worsened the inflammatory burden [202,203]. These results were also confirmed by using brodalumab, a human monoclonal antibody targeting IL-17RA [204]. However, these negative results led to the observation that IL-17 is a really important cytokine for intestinal homeostasis, wound repair, and barrier function [205,206].

Taken together, these studies indicate that our understanding for cytokine functions and its downstream mediators has significantly increased over the years. Cytokine blockade or augmentation will remain a crucial strategy for IBD and cancer therapy. However, there is still a need to optimize specific targets and establish stable delivery systems in order to ensure effective clinical therapy in the future (Figure 5).

## 6. Conclusions

This review sheds light on cytokines as crucial moderators in the maintenance of intestinal homeostasis. The multifunctional and complex nature of cytokines enables them to participate in key regulatory mechanisms in the gut, like cellular proliferation, cell death, safe-guarding of epithelial tight junctions, maintenance of a healthy microbial flora, etc. (Table 1 and Table S1). Despite decades of scientific research in cytokine signaling by using animal models of infection, IBD and cancer are still lacking in the characterization and role of single cytokines on immune–epithelial interactions. A possible solution for this aforementioned dilemma is to utilize the organoid technology and elucidate both the secretome and proteome of epithelial cells after stimulation with cytokines or bacteria-derived metabolites. This will establish a clear connection among the individual cytokine, microbiota, immune cells, and epithelial alteration in a synchronized manner.

## Figures and Tables

**Table 1:** A list of major cytokines in intestinal health and pathology along with their specific functions.

Cytokine	Cellular Source(s)	Target Cell(s)	Function
**IL-22**	ILC, Th22, NK cell, Th1, Th17	IECs	Activation of STAT3 signaling and release of AMPs
**IL-1β**	IECs	Mϕ, Endothelial cells	Activation of T-cells and ILCs
**IL-18**	IECs	Th17, Tregs	IEC proliferation, tissue regeneration, production of proinflammatory cytokines
**IL-6**	IECs, fibroblasts, Mϕ	Th17, IELs, IECs	IEC proliferation and repair, activation of STAT3, crypt homeostasis
**IL-23**	IECs, Mϕ, DC	IELs, ILC3, NK cell, T-cells	Proinflammatory cytokine secretion contributes to chronic inflammation
**IL-12**	Monocyte, Mϕ, DC	Th1	T-cell survival and differentiation, proliferation of NK cell
**IL-17A**	Th1, ILC3	IECs	Antimicrobial response, maintenance of homeostasis
**TNF**	ILC1, Mϕ	IECs	Epithelial cell death, epithelial cell migration during wound healing, mucosal repair during inflammation
**INF-γ**	ILC1, Mϕ	IECs, DC, Tregs	Confers protection against pathogens, activation of STAT1 signaling, disruption of epithelial barrier
**TGF-β**	IECs, DC, Tregs, Mast cells	B-cells, Th9 cells, Mϕ	Expansion of Tregs, IgA secretion, IEL development, tight junction maintenance
**IL-4**	ILC2, Th2,	IECs, Mast cells	Differentiation of IECs to secretory cells, confers protection against intestinal parasite infection, survival of malignant cells, activation of STAT6 signaling
**IL-5**	ILC2, Th2, B-cells	IECs, B-cells, Eosinophills	Differentiation of IECs to secretory cells, confers protection against intestinal parasite infection
**IL-9**	ILC2, Th9	IECs	Differentiation of IECs to secretory cells, leakiness in gut barrier
**IL-13**	ILC2, Th2	IECs	Differentiation of IECs to secretory cells, mucin production, confers protection against intestinal parasite infection, activation of STAT6 signaling
**IL-25**	IECs	ILC2, T-cells	Host protection against intestinal helminthes, type 2 immune response
**TSLP**	IECs, Mast cells, DC	Th2 cells, ILC2	Type 2 immune response, T- and B-cell activation
**IL-33**	IECs, intestinal myofiboblasts	ILC2, Tregs, Th2 cells IECs	Type 2 immune response, IEC differentiation, intestinal inflammation
**IL-7**	IECs	ILC3, Tregs, T effector cells	Proinflammatory cytokine secretion, IEC homeostasis
**GM-CSF**	T cells, ILC3	Monocyte, Mϕ, Tregs	Mϕ differentiation, IgA secretion from B-cells, bacterial clearance, epithelial repair during wound healing
**IL-2**	T cells	Th1, Tregs	Activation of STAT3/5 signaling, differentiation of T-cells, intestinal homeostasis
**IL-10**	Mϕ, Tregs	IECs	Intestinal homeostasis, IEC proliferation
**IL-11**	Mϕ	Malignant IECs	Activation of JAK/STAT signaling, tumor cell survival
**IL-15**	IECs	T-cells, IELs	Epithelial barrier disruption, antitumorigenic functions
**OSM**	T cells, DC	Stromal cells	Proinflammatory cytokine secretion, activation of JAK/STAT signaling
**Areg**	Tregs, ILC2	IECs	Tissue repair after damage, fibrosis
**VEGF**	Stromal cells, Mast cells, Platelets	IECs	Malignant cell survival, angiogenesis, intestinal stem cell proliferation

(IL—interleukin; INF—interferon; TNF—tumor necrosis factor; TGF—transforming growth factor; TSLP—thymic stromal lymphoprotein; GM-CSF—granulocyte-macrophage colony-stimulating factor; OSM—oncostatin M; Areg—amphiregulin; VEGF—vascular endothelial growth factor; AMP—antimicrobial peptides; IEC—intestinal epithelial cells; IEL—intraepithelial lymphocytes; DC—dendritic cells; Mϕ—macrophages; ILC—innate lymphoid cells).

## Supplementary Materials

The following are available online at https://www.mdpi.com/2073-4409/10/1/111/s1, Table S1: A list of major cytokines in intestinal health and pathology along with their specific functions.
