# Supplementary material for: Cytokine-Mediated Crosstalk between Immune Cells and Epithelial Cells in the Gut
Source: Cells. 2021 Jan 9;10(1):111. doi: 10.3390/cells10010111 (PMC7827439; doi:10.3390/cells10010111)
Supplement: Supplementary file 1 [file cells-10-00111-s001.pdf]

Table S1. A list of major cytokines in intestinal health and pathology along with their specific functions (IL—Interleukin; INF—Interferon; TNF—Tumor necrosis factor; TGF—transforming growth factor; TSLP—Thymic stromal lymphoprotein; GM-CSF—Granulocyte-macrophage colony-stimulating factor; OSM—Oncostatin M; Areg—Amphiregulin; VEGF—Vascular endothelial growth factor; AMP—anti microbial peptides; IEC—Intestinal epithelial cells; IEL—Intra-epithelial lymphocytes; DC—Dendritic cells; Mϕ—Macrophages; ILC—innate lymphoid cells).

Cytokine	Cellular Source(s)	Target cell (s)	Function
IL-22	ILC, Th22, NK cell, Th1, Th17	IECs	Activation of STAT3 signaling and release of AMPs
IL-1β	IECs	Mϕ, Endothelial cells	Activation of T cells and ILCs
IL-18	IECs	Th17, Tregs	IEC proliferation, tissue regeneration, production of pro-inflammatory cytokines
IL-6	IECs, fibroblasts, Mϕ	Th17, IELs, IECs	IEC proliferation and repair, activation of STAT3, crypt homeostasis
IL-23	IECs, Mϕ, DC	IELs, ILC3, NK cell, T cells	Pro-inflammatory cytokine secretion,contributes to chronic inflammation
IL-12	Monocyte, Mϕ, DC	Th1	T cell survival and differentiation, proliferation of NK cell
IL-17A	Th1, ILC3	IECs	Anti-microbial response, maintainence of homeostasis
TNF	ILC1, Mϕ	IECs	Epithelial cell death, epithelial cell migration during wound healing, mucosal repair during inflammation
INF-γ	ILC1, Mϕ	IECs, DC, Tregs	Confers protection against pathogens, activation of STAT1 signaling, disruption of epithelial barrier
TGF-β	IECs, DC, Tregs, Mast cells	B cells, Th9 cells, Mϕ	Expansion of Tregs, IgA secretion, IEL development, tight junction maintainence
IL-4	ILC2, Th2	IECs, Mast cells	Differentiation of IECs to secretory cells, confers protection against intestinal parasite infection, survival of malignant cells, activation of STAT6 signaling
IL-5	ILC2, Th2, B cells	IECs, B cells, Eosinophills	Differentiation of IECs to secretory cells, confers protection against intestinal parasite infection
IL-9	ILC2, Th9	IECs	Differentiation of IECs to secretory cells, leakiness in gut barrier
IL-13	ILC2, Th2	IECs	Differentiation of IECs to secretory cells, mucin production, confers protection against intestinal parasite infection, activation of STAT6 signaling
IL-25	IECs	ILC2, T cells,	Host protection against intestinal helminthes, Type 2 immune response
TSLP	IECs, Mast cells, DC	Th2 cells, ILC2	Type 2 immune response, T and B cell activation
IL-33	IECs, intestinal myofiboblasts	ILC2, Tregs, Th2 cells IECs	Type 2 immune response, IEC differentiation , intestinal inflmmation
IL-7	IECs	ILC3, Tregs, T effector cells	Pro-inflammatory cytokine secretion, IEC homeostasis
GM-CSF	T cells, ILC3	Monocyte, Mϕ, Tregs	Mϕ differentiation, IgA secretion from B cells, bacterial clearance, epithelial repair during wound healing
IL-2	T cells	Th1, Tregs	Activation of STAT3/5 signaling, differentiation of T cells, Intestinal homeostasis
IL-10	Mϕ, Tregs	IECs	Intestinal homeostasis, IEC proliferation,
IL-11	Mϕ	Malignant IECs	Activation of JAK/STAT signaling, tumor cell survival
IL-15	IECs	T cells, IELs	Epithelial barrier disruption, anti-tumorigenic functions
OSM	T cells, DC	Stromal cells	Pro-inflammatory cytokine secretion, activation of JAK/STAT signaling
Areg	Tregs, ILC2	IECs	Tissue repair after damage, fibrosis
VEGF	Stromal cells, Mast cells, Platelets	IECs	Malignant cell survival, angiogenesis, intestinal stem cell proliferation
